# Outcomes in Critically Ill Patients Sedated with Intravenous Lormetazepam or Midazolam: A Retrospective Cohort Study

**DOI:** 10.3390/jcm10184091

**Published:** 2021-09-10

**Authors:** Björn Weiss, David Hilfrich, Gerald Vorderwülbecke, Maria Heinrich, Julius J. Grunow, Nicolas Paul, Jochen Kruppa, Bruno Neuner, Berthold Drexler, Felix Balzer, Claudia D. Spies

**Affiliations:** 1 Department of Anesthesiology and Intensive Care Medicine (CCM, CVK), Charité—Universitätsmedizin Berlin, Corporate Member of Freie Universität Berlin and Humboldt Universität zu Berlin, 13353 Berlin, Germany; david.hilfrich@charite.de (D.H.); gerald.vorderwuelbecke@charite.de (G.V.); maria.heinrich@charite.de (M.H.); julius.grunow@charite.de (J.J.G.); nicolas.paul@charite.de (N.P.); bruno.neuner@charite.de (B.N.); claudia.spies@charite.de (C.D.S.); 2 Berlin Institute of Health at Charité—Universitätsmedizin Berlin, 10117 Berlin, Germany; 3 Institute of Medical Informatics, Charité—Universitätsmedizin Berlin, Corporate Member of Freie Universität Berlin and Humboldt Universität zu Berlin, 10117 Berlin, Germany; jochen.kruppa@charite.de (J.K.); felix.balzer@charite.de (F.B.); 4 Department of Anesthesiology and Intensive Care, Experimental Anesthesiology Section, Eberhard Karls Universität Tübingen, 72076 Tübingen, Germany; berthold.drexler@uni-tuebingen.de

**Keywords:** benzodiazepines, intensive care, lormetazepam, midazolam, mortality, sedation, sedation index, survival

## Abstract

The benzodiazepine, midazolam, is one of the most frequently used sedatives in intensive care medicine, but it has an unfavorable pharmacokinetic profile when continuously applied. As a consequence, patients are frequently prolonged and more deeply sedated than intended. Due to its distinct pharmacological features, including a cytochrome P450-independent metabolization, intravenous lormetazepam might be clinically advantageous compared to midazolam. In this retrospective cohort study, we compared patients who received either intravenous lormetazepam or midazolam with respect to their survival and sedation characteristics. The cohort included 3314 mechanically ventilated, critically ill patients that received one of the two drugs in a tertiary medical center in Germany between 2006 and 2018. A Cox proportional hazards model with mortality as outcome and APACHE II, age, gender, and admission mode as covariates revealed a hazard ratio of 1.75 [95% CI 1.46–2.09; *p* < 0.001] for in-hospital mortality associated with the use of midazolam. After additionally adjusting for sedation intensity, the HR became 1.04 [95% CI 0.83–1.31; *p* = 0.97]. Thus, we concluded that excessive sedation occurs more frequently in critically ill patients treated with midazolam than in patients treated with lormetazepam. These findings require further investigation in prospective trials to assess if lormetazepam, due to its ability to maintain light sedation, might be favorable over other benzodiazepines for sedation in the ICU.

## 1. Introduction

The use of sedatives is an essential element of intensive care medicine for stress and anxiety reduction as well as tolerance for invasive therapies such as mechanical ventilation [1,2]. However, a growing body of evidence indicates that deep sedation is a risk factor for prolonged mechanical ventilation [3] as well as increased mortality and morbidity in critically ill patients [4,5,6]. 

As sedation intensity shows a dose-dependent effect on 180-days mortality [7], avoiding deep sedation while treating specific symptoms such as anxiety and stress has become a desirable and necessary feature of a sedative agent in the intensive care unit (ICU) [2].

Benzodiazepines, which act as positive allosteric modulators at the GABA_A_ receptor, are commonly used for sedation and anxiolysis in the ICU, but they have been criticized for causing prolonged deep sedation and delirium [8]. Due to its particularly broad therapeutic range and good hemodynamic stability, midazolam is the most frequently used benzodiazepine in critical care [9]. However, its continuous application is under particular criticism for prolonged deep sedation [2,10,11]. Reasons for this include the metabolization and clearance of the substance: Midazolam undergoes a cytochrome P450-mediated biotransformation, and accumulation of its metabolite, 1-hydroxymidazolam, amplifies midazolam’s sedative effect, particularly if continuously applied [12]. It was shown that a continuous midazolam infusion for three days significantly elevates plasma levels of active metabolites for weeks [11]. This corresponds to clinical trials reporting prolonged awakening after benzodiazepine infusion [13,14].

In 2008, the benzodiazepine lormetazepam was repurposed as an intravenous formulation for sedation in Germany. The substance is metabolized and cleared independently from the cytochrome P450 system, and its metabolites are inactive [15].

Since 2010, both substances have been available and could be administered for the same purposes in the ICU. The use of either midazolam or lormetazepam depends on the physicians’ decision and standard operating procedures. The concomitant use of both substances offers the opportunity to conduct a retrospective analysis comparing midazolam and lormetazepam in routine use.

To our knowledge, midazolam and lormetazepam have not been compared with respect to sedation depth and mortality risk. We hypothesize that patients treated with lormetazepam are more likely to achieve a state of no or light sedation and, thus, are more likely to survive. The explorative analysis aims to generate hypotheses to assess lormetazepam in randomized controlled trials.

## 2. Materials and Methods

### 2.1. Study Design and Ethics

We report a retrospective observational cohort study conducted at Charité—Universitätsmedizin Berlin, Germany. Ethical approval was obtained from the institutional review board of Charité—Universitätsmedizin Berlin, Germany (EA2/075/019). Informed consent was waived. The study was prospectively registered at ClinicalTrials.gov (NCT04408911).

### 2.2. Data Sources

Routine data were collected from the hospital’s electronic health record (SAP, Walldorf, Germany) and ICU patient data management system (COPRA, Sasbachwalden, Germany). All identifiable information and case-specific numbers were removed before the analysis.

### 2.3. Patient Selection and Inclusion Criteria

Patients admitted to all medical and surgical ICUs at Charité—Universitätsmedizin Berlin, Germany, between 1 January, 2006 and 31 December, 2018 were screened for eligibility. We did not screen neuro-ICUs, because in neuro-critically ill patients, the indications for benzodiazepines differ from standard operating procedures of medical and surgical ICUs. Inclusion criteria were as follows: age ≥ 18 years; mechanical ventilation; patient received either midazolam or lormetazepam; and ICU length of stay ≥48 h. Exclusion criteria were: application of both, midazolam and lormetazepam, and application of only a single bolus of midazolam or lormetazepam during the entire ICU stay.

### 2.4. Patient Grouping and Sedation Practice

Patients were grouped based on treatment with midazolam or lormetazepam for sedation, either as boluses or as continuous infusion. Both substances were administered according to current German clinical guidelines for the management of sedation, pain and delirium [2]. One key recommendation is that sedation is confined to concrete and special indications, and there is the overall aim to target no measurable sedation (Richmond Agitation-Sedation Scale (RASS) 0/−1) in critically ill patients. Sedation was measured with the RASS. The RASS is a ten-point scale with positive values for agitation and negative values for a reduced level of arousal, reaching from −1 (ability to open eyes and sustained eye contact for at least ten seconds) to −5 (no reaction to verbal or tactile stimulus). The RASS has been evaluated to be the most valid and reliable assessment tool for measuring sedation depth in critically ill patients according to the psychometric testing of the US guideline on pain, agitation and delirium in 2013 [16]. It is widely used and part of the routine sedation management at the study site.

During the observation period, the German guideline underwent two updates, which did not change the indication spectrum of or recommendation for benzodiazepines [2]. Sedation management was performed according to the internal standard operating procedures of the study site. 

### 2.5. Primary and Secondary Outcome Variables

The primary endpoint was in-hospital mortality. Secondary endpoints were incidence of delirium, duration of delirium, ICU length of stay, hospital length of stay, duration of mechanical ventilation, and sedation-specific characteristics. Screening of pain, agitation and delirium was performed according to the German Guidelines for the Management of Sedation, Pain and Delirium [2]. Hence, routine sedation measurement was performed at least once per shift with the RASS by the bedside nurse.

### 2.6. Data Analysis and Statistics

Sedation intensity was quantified with the sedation index (SI), which was calculated as previously described [7]. The SI is the modulus of the sum of all negative RASS assessments in a time period, divided by the number of RASS assessments in the same time period. For example, if a patient shows RASS scores of −2, −2, +1, and 0 in a 24 h period, the SI would be equal to 1. As only negative RASS scores add to the SI and all positive values are normalized to zero, the sedation index quantifies sedation depth. The underlying idea is that agitation (indicated by a positive RASS value) cannot compensate for sedation (indicated by a negative RASS value).

Deeper sedation results in higher SIs. If not indicated differently, we used the SI for 48 h after commencement of sedation with either midazolam or lormetazepam. As the SI is a measure of sedation over time, it was only calculated if more than two RASS assessments per 24 h were available.

For descriptive statistics, continuous variables were displayed as mean and standard deviation (SD) or as median and interquartile range (IQR). Categorical variables were displayed as absolute (*n*) and relative (%) frequencies. The midazolam and lormetazepam groups were compared regarding baseline characteristics, sedation characteristics, and outcome parameters using unpaired t-test, Mann–Whitney U-test, Fisher’s exact test, and χ^2^ test. To analyze the mortality risk between the midazolam group and lormetazepam group, we fitted multivariable logistic regression models that were adjusted for Acute Physiology and Chronic Health Evaluation (APACHE) II score, age, gender, admission due to medical reasons, and admission due to emergency surgery. We further analyzed the mortality using Kaplan–Meier plots for the midazolam group and lormetazepam group, and both groups stratified by sedation levels (light sedation and deep sedation). Kaplan–Meier curves were compared using log-rank tests. After checking the proportional hazard assumption, we fitted two Cox proportional hazards models with death as outcome. Model 1 included the use of midazolam (yes/no), APACHE II, age, gender, and admission mode as covariates. Model 2 additionally adjusted for a SI ≥ 1.5 in the first 48 h (yes/no). We defined statistical significance at *p* < 0.05. Analyses were carried out using SPSS (version 26) and R (version 4.0.5).

## 3. Results

### 3.1. Patient Characteristics

**Figure 1:**
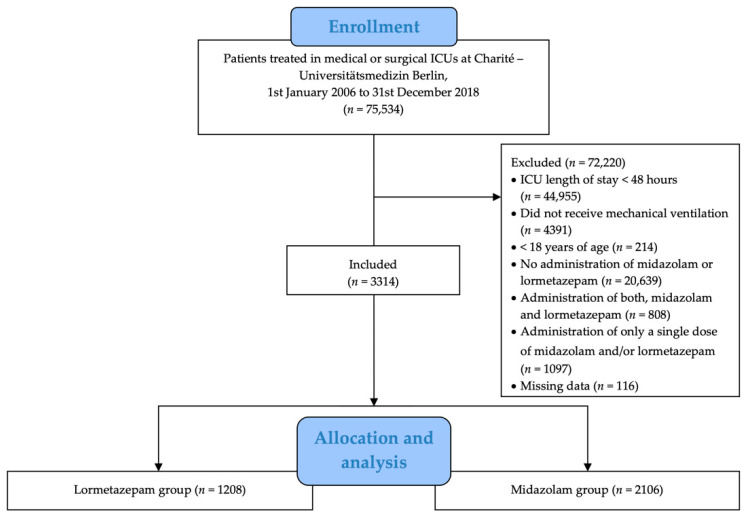
Inclusion and exclusion diagram.

Out of 75,534 patients screened for eligibility, 3314 patients were included in the analysis (Figure 1). Most patients were excluded due to an ICU length of stay of <48 h, no administration of either midazolam or lormetazepam, or no mechanical ventilation.

Patients in the midazolam group had a significantly higher APACHE II score (19.6 (SD 9.6) vs. 23.6 (SD 9.8); *p* < 0.001) indicating a higher severity of illness, and were more likely to have been admitted to the ICU due to medical reasons (44% vs. 38%; *p* < 0.001; Table 1). Preexisting delirium was more common in the lormetazepam group (75% vs. 36%; *p* < 0.001).

### 3.2. Sedation Characteristics

In 84% of cases, lormetazepam was administered as a bolus, and in about one third of cases as a continuous infusion (Table 2). In contrast, only one of four patients received midazolam as a bolus, and about nine of ten patients received midazolam as a continuous infusion. Patients receiving midazolam had a significantly lower SI in the first 48 h after administration of the substance (mean SI 4.10 (SD 1.0) vs. 1.7 (SD 1.5)) and were more deeply sedated (median RASS −4 (IQR −5; −3) vs. 0 (IQR −1; +1)). The difference in SI between the midazolam group and the lormetazepam group was consistent over the observation period, even though both groups showed a continuous trend towards higher SIs from 2006 to 2018 (Table 3*).*

### 3.3. Primary Outcome Measure

In-hospital mortality was significantly higher in the midazolam group than in the lormetazepam group (23% vs. 42%; *p* < 0.001; Table 4). In multivariable logistic regression analysis, and adjusted for APACHE II, age, gender, and admission due to medical reasons or emergency surgery, midazolam was associated with higher odds for in-hospital mortality compared to lormetazepam (odds ratio (OR) = 2.04 (95% confidence interval (95% CI) 1.71–2.45; *p* < 0.001; Table 5).

**Figure 2:**
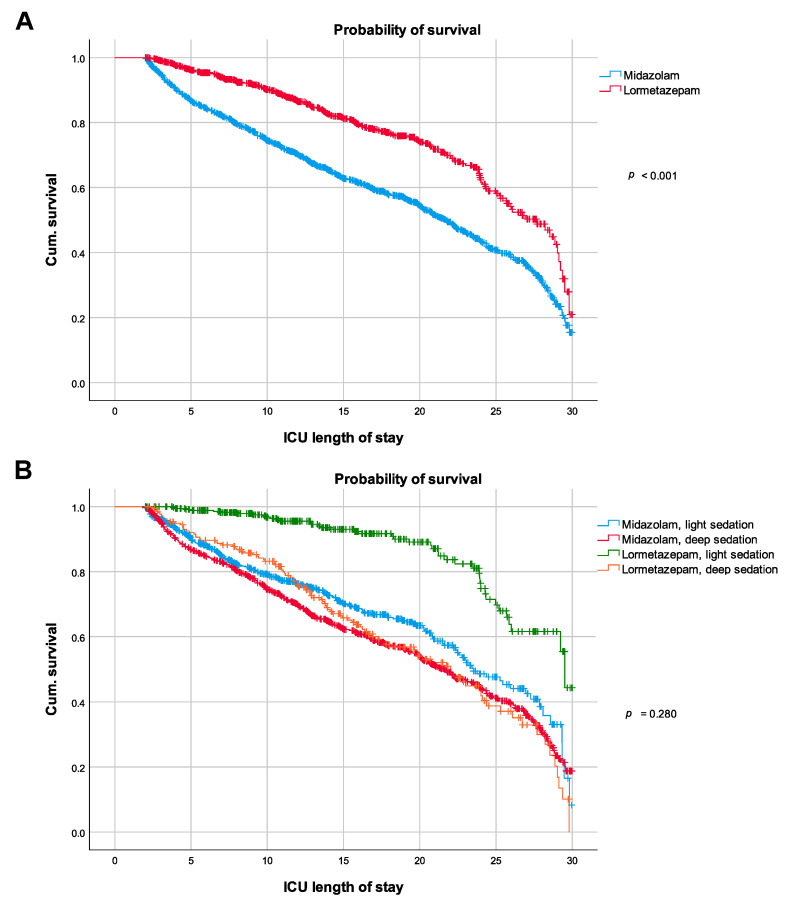
Kaplan–Meier curves of (**A**) the midazolam and lormetazepam groups, and (**B**) the midazolam and lormetazepam groups, stratified by sedation depth. Survival probability between groups was compared using the log-rank test. Cum. survival, cumulative survival; ICU, intensive care unit.

Using the Kaplan–Meier method, the lormetazepam group shows a higher survival probability compared with the midazolam group (Figure 2A; log-rank test: *p* < 0.001). Confirming the findings from the Kaplan–Meier analysis, the Cox proportional hazards model for the in-hospital mortality with APACHE II, age, gender, and admission mode as covariates revealed a hazard ratio (HR) of HR = 1.75 (95%-CI 1.46–2.09; *p* < 0.001) for the use of midazolam (Table 6, Model 1). However, when additionally accounting for the differences in sedation depth by adding the SI to the model (SI ≥ 1.5 in the first 48 h, yes/no), the effect of midazolam disappeared (HR = 1.04 (95%-CI 0.83–1.31); *p* = 0.97; Table 6, Model 2). Inclusion of the SI as a continuous variable resulted in the same outcome (Supplementary Table S1). When we stratified the midazolam group and lormetazepam group for light and deep sedation, we did not find significant differences in the survival probability (Figure 2B).

### 3.4. Secondary Outcomes

Patients in the midazolam group stayed significantly longer in the ICU (mean 31.5 days (SD 28.7) vs. 24 days (SD 23.1); *p* < 0.001) and in the hospital (mean 49.7 days (SD 43.1) vs. 40.6 days (SD 36.8); *p* < 0.001) than in the lormetazepam group (Table 4). In addition, patients in the midazolam group were longer mechanically ventilated than patients in the lormetazepam group (mean 606.9 h (SD 633.9) vs. 520.7 h (SD 712.1); *p* = 0.004).

## 4. Discussion

In this retrospective cohort study, we found that the intravenous benzodiazepine lormetazepam is associated with a lower in-hospital mortality when compared to midazolam in adult critically ill patients. The effect completely disappeared when correcting for sedation intensity. 

We are not aware of any study that showed an increased risk of mortality in critically ill patients treated with midazolam when compared to other substances. In our study, we observed an increased mortality risk in patients treated with midazolam in the univariate and multivariate analyses. However, this effect depended on sedation intensity; when the SI was added to the Cox proportional hazards model, the effect on the mortality risk disappeared. Patients being treated with midazolam seemed only more likely to have a fatal outcome because they were more deeply sedated. This does not come by surprise, as early deep sedation has been identified as a risk factor for mortality in critically ill patients in several studies [4,6,7].

To our knowledge, this is the first study comparing the repurposed benzodiazepine lormetazepam to a traditional benzodiazepine. Previous studies on benzodiazepines in critically ill patients have mostly compared midazolam and lorazepam to the non-benzodiazepines propofol or dexmedetomidine [1,2]. In these studies, midazolam and lorazepam were associated with longer mechanical ventilation and an increased risk for delirium [8,17,18]. A meta-analysis of studies for the 2013 Clinical Practice Guidelines for the Management of Pain, Agitation, and Delirium in Adult Patients in the Intensive Care Unit revealed an ICU length of stay half of a day longer in patients receiving benzodiazepines compared to non-benzodiazepines [16].

Our surprising findings question current guideline and expert recommendations that suggest to refrain from the general use of benzodiazepines in favor of non-benzodiazepines [1,2,19,20]. Subsuming all benzodiazepines in one group is based on the assumption that they act similarly, but our results allow the hypothesis that this might be an oversimplification, as there are relevant differences between benzodiazepines [21]. A more nuanced perspective, which considers the advantages and disadvantages of each substance, seems a promising, new approach.

There is a potential explanation from experimental research that suggests that the detrimental effects of midazolam might be attributable to its pharmacokinetic properties and metabolization. For conversion to a water-soluble product, midazolam undergoes two phases of biotransformation, involving cytochrome P450-dependent hydroxylation and glucuronidation [14,22,23]. The first metabolite, 1-hydroxymidazolam, is still active and can contribute to the sedative effect of midazolam, especially when 1-hydroxymidazolam accumulates in continuous applications [12]. Following this hypothesis, the use of midazolam is related to deep sedation if delivered in a continuous infusion [11]. With midazolam, sedation might not only be deeper but also more difficult to regulate compared to sedation with other benzodiazepines. Our results reflect these findings; patients that were treated with midazolam had deeper and prolonged sedation. In contrast to midazolam, lormetazepam does not undergo phase-one biotransformation, but is directly glucuronidated, which might make its half-life more predictable. In addition, there is no known active metabolite of lormetazepam [15]. Both aspects of lormetazepam might contribute to a better regulation of sedation depth, which we also observed in our analysis. This might also be an explanation why physicians chose a bolus-wise application of lormetazepam more often than for midazolam.

One has to consider several limitations when interpreting this study. Firstly, this is a monocentric, retrospective observational study that can only be considered hypothesis-generating. A change of sedation practice over the observation period or a difference in sedation practice between the groups might have impacted our results. To investigate if there was a change in sedation levels over time, we analyzed the mean SIs for each year of the observation period and detected a steady increase in mean SIs for both, the midazolam and lormetazepam group. This might be because we generally use fewer sedatives, but when we use them, there is a medical indication for a deeper sedation. In summary, there is a profound difference between sedation depths in the midazolam and the lormetazepam group that cannot be conclusively explained because of the uncontrolled, retrospective nature of our study. Moreover, the SI could not be calculated for about one quarter of patients in both groups due to too few documented RASS assessments within 48 h after commencement of sedation. This might have introduced a systematic bias in our analysis. In addition, we only analyzed midazolam and lormetazepam, neglecting other co-sedatives and other administered medications that might have acted as confounders by affecting SIs and mortality. In addition, dosages of sedatives were not available, but we are certain that substances were used for sedation purposes in general ICU patients according to the manufacturers’ dosages. Benzodiazepines were typically applied according to the German guidelines after an initial phase of propofol, which was never applied for more than seven days due to legal restrictions [2]. 

Findings of this study should be confirmed in a randomized controlled trial. Furthermore, future studies comparing midazolam and lormetazepam should take the administration of alpha-2 agonists, analgesics, neuroleptics, and additional sedatives into consideration.

## 5. Conclusions

In adult critically ill patients, decreased survival was associated with deeper sedation which is in line with previous studies. We found that in the midazolam compared to the lormetazepam group, deep sedation occurred more frequently. Differences in sedation depth and mortality between patients receiving lormetazepam and midazolam should be compared in a prospective, randomized controlled trial, which should have a particular focus on the ability of the substances to maintain a light-sedation regime in the ICU.

## Figures and Tables

**Table 1:** Baseline characteristics of patients in the midazolam group and lormetazepam group.

Baseline Characteristic	Lormetazepam (*n* = 1208)	Midazolam (*n* = 2106)	*p*
Age, years ^a^	61.2 (16.1)	61.6 (15.9)	0.49
Female sex, *n* (%)	406 (34%)	700 (33%)	0.83
BMI, kg/m^2 a^	27.0 (7.1)	27.3 (6.6)	0.41
APACHE II ^a^	19.8 (9.6)	23.6 (9.8)	<0.001
Charlson’s comorbidity index ^a^	5.6 (3.4)	5.8 (3.1)	0.3
Preexisting delirium, *n* (%)	903 (75%)	765 (36%)	<0.001
Reason of admission, *n* (%)			
Elective surgery	335 (28%)	365 (17%)	<0.001
Emergency surgery	294 (24%)	479 (23%)	
Medical	465 (38%)	933 (44%)	
No data	114 (9%)	329 (16%)	

^a^ Mean (standard deviation). APACHE II, Acute Physiology and Chronic Health Evaluation II score; BMI, body mass index.

**Table 2:** Sedation-related characteristics of patients in the midazolam group and lormetazepam group.

Characteristic	Lormetazepam (*n* = 1208)	Midazolam (*n* = 2106)	*p*
Sedation index for the first 48 h after ICU admission ^a^	1.7 (1.5)	4.10 (1.0)	<0.001
Sedation index ≥ 1.5 in first 48 h, *n* (% of non-missing)	355 (40.4%) ^d^	1506 (95.9%) ^d^	<0.001
Sedation index < 1.5 in first 48 h, *n* (% of non-missing)	523 (59.6%) ^d^	64 (4.1%) ^d^	
RASS ^b^	0 (−1; 0.5)	−4 (−5; −3)	<0.001
Total rate of delirium, *n* (%)	837 (69%)	677 (32%)	<0.001
Any bolus administration ^c^, *n* (%)	1010 (84%)	518 (25%)	<0.001
Any continuous administration ^c^, *n* (%)	433 (36%)	1873 (89%)	<0.001

^a^ Mean (standard deviation). ^b^ Median (25th percentile; 75th percentile). ^c^ Administration on at least one occasion. ^d^ For *n* = 330 patients in the lormetazepam group and *n* = 536 in the midazolam group, sedation index could not be calculated due to too few RASS assessments within 48 h. ICU, intensive care unit; RASS, Richmond Agitation Sedation Scale.

**Table 3:** Mean sedation index by sedative used and year of treatment.

		Sedation Index by Sedative *			
		Midazolam		Lormetazepam	
		*n*	Mean	*n*	Mean
Year	2006	114	3.412	0	
	2007	119	3.654	0	
	2008	173	3.799	3	0.889
	2009	227	4.027	10	0.542
	2010	290	4.221	6	0.125
	2011	285	4.190	19	0.591
	2012	265	4.279	44	0.668
	2013	158	4.292	60	1.458
	2014	145	4.077	103	1.878
	2015	92	3.884	190	1.624
	2016	66	4.374	278	1.491
	2017	61	4.443	264	1.863
	2018	111	4.067	231	1.848

Table showing number of patients treated each year with lormetazepam or midazolam and the respective average sedation index in the first 48 h after initiation of treatment with one of the substances. * Sedation index in the first 48 h after initiation of sedative treatment.

**Table 4:** Univariate comparison of outcome parameters for the midazolam group and the lormetazepam group.

Variable	Lormetazepam (*n* = 1208)	Midazolam (*n* = 2106)	*p*
Hospital mortality, *n* (%)	276 (23%)	883 (42%)	<0.001
Duration of mechanical ventilation, hours ^a^	520.7 (712.1)	606.9 (633.9)	0.004
ICU length of stay, days ^a^	24 (23.1)	31.5 (28.7)	<0.001
Hospital length of stay, days ^a^	40.6 (36.8)	49.7 (43.1)	<0.001

^a^ Mean (standard deviation). ICU, intensive care unit.

**Table 5:** Multivariable logistic regression for in-hospital mortality.

Variable	Odds Ratio (95% Confidence Interval) for In-Hospital Mortality	*p*
Use of midazolam, yes/no	2.04 (1.71–2.45)	<0.001
APACHE II	1.03 (1.02–1.04)	<0.001
Age, years	1.01 (1.01–1.02)	<0.001
Gender, female	1.15 (0.97–1.37)	0.11
Cause of admission, medical	1.23 (0.99–1.53)	0.06
Cause of admission, emergency surgery	0.89 (0.69–1.14)	0.35

APACHE II, Acute Physiology and Chronic Health Evaluation II score.

**Table 6:** Cox proportional hazards models for the in-hospital mortality.

Variable	Hazard Ratio (95% Confidence Interval) for In-Hospital Mortality	*p*
Model 1		
Use of midazolam, yes/no	1.75 (1.46–2.09)	<0.001
Age, years	1.01 (1.01–1.02)	<0.001
Gender, female	1.07 (0.91–1.25)	0.43
APACHE II	1.03 (1.02–1.04)	<0.001
Emergency surgery, yes/no	0.77 (0.61–0.98)	0.04
Model 2		
Use of midazolam, yes/no	1.04 (0.83–1.31)	0.97
Sedation index ≥ 1.5 in the first 48 h, yes/no	3.14 (2.23–4.43)	<0.001
Age, years	1.01 (1.01–1.02)	<0.001
Gender, female	1.04 (0.87–1.26)	0.46
APACHE II	1.03 (1.02–1.04)	<0.001
Emergency surgery, yes/no	0.72 (0.54–0.94)	0.02

Model 1 is adjusted for midazolam use (yes/no), age, APACHE II, and emergency surgery (yes/no). Model 2 is adjusted for the same covariates plus the sedation index. APACHE II, Acute Physiology and Chronic Health Evaluation II score.

## Data Availability

The data presented in this study are available on request from the corresponding author. The data are not publicly available due to local data protection requirements.

## Supplementary Materials

The following are available online at https://www.mdpi.com/article/10.3390/jcm10184091/s1, Table S1: Cox proportional hazards model including the sedation index as continuous variable.
