# Supplementary material for: Outcomes in Critically Ill Patients Sedated with Intravenous Lormetazepam or Midazolam: A Retrospective Cohort Study
Source: J Clin Med. 2021 Sep 10;10(18):4091. doi: 10.3390/jcm10184091 (PMC8465285; doi:10.3390/jcm10184091)
Supplement: Supplementary file 1 [file jcm-10-04091-s001.zip › jcm-1354943-supplementary.pdf]

Journal of
Clinical Medicine

Table S1. Cox proportional hazards models.

	Hazard Rate<br>(95% CI) for ICU Length of Stay in Days	*p*
Cox regression including APACHE II, age, gender and admission mode:		
Use of midazolam	1.75 (1.46–2.09)	< 0.001
Age	1.01 (1.01–1.02)	< 0.001
APACHE II	1.03 (1.02–1.04)	< 0.001
Emergency surgery	0.77 (0.61–0.98)	0.036
Cox regression with additional inclusion of sedation index:		
Use of midazolam	1.04 (0.83–1.31)	0.973
SI48 ≥ 1,5	3.14 (2.23–4.43)	< 0.001
Age	1.01 (1.01–1.02)	< 0.001
APACHE II	1.03 (1.02–1.04)	< 0.001
Emergency surgery	0.72 (0.54–0.94)	0.017

Cox proportional hazards model of mortality during the intensive care unit stay. ICU: Intensive care unit; APACHE II: Acute physiology and chronic health evaluation II; SI48: Sedation index in the first 48 hours after initiation of treatment.
